# Regulation of Cell Proliferation and Differentiation by PPAR*β*/*δ*

**DOI:** 10.1155/2008/614852

**Published:** 2008-09-16

**Authors:** Rolf Müller, Markus Rieck, Sabine Müller-Brüsselbach

**Affiliations:** Institute of Molecular Biology and Tumor Research (IMT), Philipps-University, Emil-Mannkopff-Strasse 2, 35032 Marburg, Germany

## Abstract

Peroxisome proliferator-activated receptor-*β*/*δ* (PPAR*β*/*δ*) is a ligand-activated transcription factor with essential functions in the regulation of lipid catabolism, glucose homeostasis, and inflammation, which makes it a potentially relevant drug target for the treatment of major human diseases. In addition, there is strong evidence that PPAR*β*/*δ* modulates oncogenic signaling pathways and tumor growth. Consistent with these observations, numerous reports have clearly documented a role for PPAR*β*/*δ* in cell cycle control, differentiation, and apoptosis. However, the precise role of PPAR*β*/*δ* in tumorigenesis and cell proliferation remains controversial. This review summarizes our current knowledge and proposes a model corroborating the discrepant data in this area of research.

## 1. INTRODUCTION

Peroxisome proliferator-activated receptor-*β*/*δ* (PPAR*β*/*δ*) is a transcription factor that is activated by endogenous fatty acid ligands and by synthetic agonists [1, 2]. Major functions of PPAR*β*/*δ* are associated with the regulation of glucose, energy, and lipid metabolism [3], and the control of inflammatory responses [4, 5]. PPAR*β*/*δ*, therefore, represents a promising drug target for the treatment of common diseases such as obesity, metabolic syndrome, chronic inflammation, and arteriosclerosis, which has led to the development of synthetic drug agonists with subtype selectivity and high-affinity binding [6]. Mice lacking PPAR*β*/*δ* show an aberrant development of the placenta and exhibit a defect in wound healing associated with alterations in cell proliferation, differentiation, and cellular survival [7–10]. Experimental evidence obtained with cultured cells has provided additional strong evidence for a role of PPAR*β*/*δ* in cell cycle regulation and differentiation in different cell types (see Table 1). Consistent with these physiological functions, there is also clear evidence for a role of PPAR*β*/*δ* in oncogenesis and tumor growth. These findings might provide a basis for the development of novel strategies for the treatment of proliferative diseases, but also demand some caution with respect to the clinical use of PPAR*β*/*δ*-directed dugs. A detailed knowledge of the role of PPAR*β*/*δ* in cell proliferation and its effects on tumor growth are therefore of paramount importance.

## 2. PPAR*β*/*δ* AFFECTS TUMORIGENESIS

The role of PPAR*β*/*δ* in tumorigenesis has been explored predominantly in epithelial tumors of the skin, lung, and intestine and in the tumor stroma. PPAR*β*/*δ* inhibits chemically induced skin carcinogenesis, since an enhancement of chemically induced skin tumor growth is seen in mice with a global disruption of *Pparb* [38]. However, no effect on skin carcinogenesis is observed in mice lacking PPAR*β*/*δ* specifically in basal keratinocytes [39], suggesting that the tumor suppressive effect of PPAR*β*/*δ* is due to a function in other cell types. A tumor suppressive role for PPAR*β*/*δ* has also been described for a transgenic mouse model of Raf oncogene-induced lung adenoma formation, but similar to skin carcinogenesis the precise mechanisms and cell types involved are not known [40]. Effects of PPAR*β*/*δ* have also been reported in different mouse models of intestinal carcinogenesis, that is, the Apc/Min mouse lacking functional APC protein and chemically induced intestinal carcinogenesis, but these studies differ in their conclusions [41]. Thus, PPAR*β*/*δ* has been reported to have either no effect on intestinal tumorigenesis [9] to attenuate tumor growth by promoting terminal differentiation of colonocytes [33, 42–45] or to potentiate tumorigenesis [46–48]. The reason for these discrepancies remains unclear at present [49], but may be in part related to a function of PPAR*β*/*δ* in host cells recruited by the tumor, such as endothelial cells, fibroblasts, and macrophages [50]. Indeed, recent work showed that PPAR*β*/*δ* is indispensable for the formation of functional tumor microvessels [29, 51], suggesting that PPAR*β*/*δ* may have different functions in the tumor stroma and in tumor cells with opposing effects on tumor growth. The role of PPAR*β*/*δ* in tumor stroma cells is further discussed below.

## 3. ATTENUATION OF TUMOR STROMA CELL PROLIFERATION BY PPAR*β*/*δ*

The inhibition of syngeneic tumor growth in mice lacking PPAR*β*/*δ* strongly correlates with a lower density of functional tumor microvessels [29, 51], which is associated with a striking increase in the proliferation of tumor endothelial cells and an inhibition of their maturation [29]. The immature microvascular structures are also frequently surrounded by perivascular cells expressing vast amounts of *α*-smooth muscle actin, giving rise to an overall picture characteristic of tumor endothelial hyperplasia. In vivo microarray analysis led to the identification of PPAR*β*/*δ* target genes with known inhibitory functions in angiogenesis, including *Cd36* and *Cdkn1c* [29]. A crucial function of CD36 is to serve as a receptor for thrombospondins which are known to attenuate the proliferation of endothelial cells [52], and *Cdkn1c* codes for the cyclin-dependent kinase inhibitor p57^KIP2^ [53]. Consistent with the existence of a PPAR*β*/*δ* – p57^KIP2^ pathway in stroma cell types, it was shown that the forced expression of PPAR*β*/*δ* in *Pparb* null fibroblasts results in a *Cdkn1c*-dependend inhibition of cell proliferation [29]. Other PPAR*β*/*δ* target genes with potential functions in cell proliferation and differentiation were identified in the same study, suggesting that PPAR*β*/*δ* regulates multiple genes with functions in cell proliferation in the context of tumor stroma development and tumor angiogenesis.

An antiproliferative effect of PPAR*β*/*δ* agonists in fibroblasts and vascular smooth muscle cells has also been observed in two other studies [27, 30], while opposite effects have been described for endothelial cells [31]. At present, it is difficult to explain these apparent discrepancies, since they cannot be narrowed down to a single parameter, such as experimental strategy, cell type, expression level of PPAR*β*/*δ*, or state of the cell (e.g., metabolic activity, proliferative status, stage of differentiation, exogenous factors). This issue is discussed further in the Conclusions section below.

## 4. ROLE OF PPAR*β*/*δ* IN WOUND HEALING AND KERATINOCYTE PROLIFERATION

*Pparb* null mice exhibit a defect in wound healing by inhibiting apoptosis in keratinocytes [8]. This survival function of PPAR*β*/*δ* has been explained by an induction of AKT/protein kinase B (PKB) activity by PPAR*β*/*δ* resulting from an upregulation of the *Pdk1* and *Ilk* genes and a downregulation of *Pten* [11]. Increased AKT signaling is generally associated with enhanced proliferation, yet others have reported that PPAR*β*/*δ* inhibits cell proliferation [7, 15]. In this case, however, AKT activity was not affected by PPAR*β*/*δ* activation. Instead, a downregulation of protein kinase C and MAP kinase signaling was observed [14]. The reason for these discrepancies is not clear at present, however, in light of the relatively small effects of PPAR*β*/*δ* on the signaling pathways discussed above it is possible that subtle differences in the experimental settings account for the apparent lack of consistency.

## 5. ROLE OF PPAR*β*/*δ* IN DIFFERENTIATION

Mice lacking PPAR*β*/*δ* show a very high degree of embryonic lethality due to an aberrant development and malfunction of the placenta [7, 9, 10]. Consistent with this finding, the differentiation and metabolic functions of trophoblast giant cells in vitro are dependent on PPAR*β*/*δ* [10]. In the same model, stimulatory effect of PPAR*β*/*δ* on AKT signaling was observed. Another tissue where PPAR*β*/*δ* plays a role in differentiation is the digestive tract, where PPAR*β*/*δ* promotes the differentiation of Paneth cells in the intestinal crypts by down-regulating the hedgehog signaling pathway [24]. A differentiation promoting effect of PPAR*β*/*δ* has also been described for keratinocytes, adipocytes, endothelial cells, and oligodendrocytes (see Table 1 for details).

## 6. CONCLUSIONS

Studies addressing the role of PPAR*β*/*δ* in differentiation have yielded a consistent picture and point to a differentiation promoting in a wide spectrum of different cell types. Numerous reports have also clearly documented a role for PPAR*β*/*δ* in cell proliferation and tumorigenesis, yet different studies have produced controversial results, even though the majority of studies describe antiproliferative effects by PPAR*β*/*δ* (see Table 1).

One reason for the apparently discrepant data may be associated with the use of different experimental strategies. Since the precise mechanisms of PPAR*β*/*δ*-mediated gene regulation are often not known, the results from gain-of-function and loss-of-function are not always easy to interpret. Thus, ligand activation and genetic inactivation of PPAR*β*/*δ* may have opposite effects, as in the case of classical PPRE-driven genes, but may also give similar results in other regulatory settings. The latter has been described, for instance, for PPAR*β*/*δ*-mediated gene repression through direct interaction with the transcriptional repressor BCL-6 in macrophages [54]. This aspect has not been thoroughly analyzed to date so that it is difficult to judge its contribution to the deviant results published in different studies.

To help explain the discrepant published data, we would therefore like to put forward another hypothesis. This model postulates that PPAR*β*/*δ* is not a *bona fide* cell cycle regulator with a defined function but rather affects the expression of both inducers and inhibitors of cell proliferation (e.g., regulators of the AKT pathway and PDGF versus the cell cycle inhibitors p57^KIP2^ and *G0S2*; see Table 1). This is conceivable both in view of the large number of potential PPAR target genes, estimated at several thousand for the human genome [55]. Depending on the particular cell type, the metabolic or proliferative state of the cell or other experimental conditions, positive or negative regulators of the cell cycle may prevail resulting in opposite effects. This suggests that the precise effects of PPAR*β*/*δ* on cell proliferation are highly context-dependent and not predictable on the basis of our current knowledge. Clearly, a better and detailed understanding of the effects of PPAR*β*/*δ* on cell cycle regulation and differentiation will be a prerequisite for the development of PPAR*β*/*δ* directed drugs and their clinical application.

## Figures and Tables

**Table 1:** Effects of PPAR*β*/*δ* on cell proliferation and differentiation.

Cell type	Exp. approach	Role of PPAR *β*/*δ* in		Affected pathway	References
		Prolif.	Diff.		
*Epithelial cells*					

Keratinocyte	Agonist, wt versus null	↘	↗	AKT	[11]
Keratinocyte	Agonist, wt versus null	↘	↗	ERK	[12–17]
Keratinocyte	Agonist, RNAi, wt versus null	↗			[18, 19]
Adipocyte	Agonist, wt versus null		↗	PPAR*γ*	[20–22]
Trophoblast	wt versus null		↗	AKT	[10, 23]
Paneth cells (in vivo)	wt versus null		↗	Hedgehog	[24]
Hepatic stellate cell	Agonist	↗			[25]
Oligodendrocyte	Agonist		↗		[26]

*Mesenchymal cells*					

Fibroblast	Agonist	↘	(↘*)	G0S2**, PTEN	[27]
Fibroblast	wt versus null, re-expression in null	↘		p57^KIP2^	[28, 29]
Vascular smooth muscle cells	Agonist	↘		PDGF	[30]
Tumor endothelium	wt versus null	↘	↗		[29]
Endothelial cells	Agonist	↗	↗		[31]

*Human tumor cell lines*					

MCF-7 breast carcinoma; UACC903 melanoma	Agonist	↘			[32]
HT29, HCT116, LS-174T colon carcinoma; HepG2, HuH7 hepatoma	Agonist	↘			[33]
HCT116 colon carcinoma	RNAi	↘			[34]
SH-SY5Y neuroblastoma	Agonist		↗		[35]
NSC lung carcinoma	Agonist	↗		AKT, NF*κ*B	[36]
A549 NSC lung ca.	Agonist	↘			[37]

*transdifferentiation into myofibroblasts.

**G0S2: *G0/G1 switch gene 2* (cell cycle inhibitor).
